# Estimating future temperature maxima in lakes across the United States using a surrogate modeling approach

**DOI:** 10.1371/journal.pone.0183499

**Published:** 2017-11-09

**Authors:** Jonathan B. Butcher, Tan Zi, Michelle Schmidt, Thomas E. Johnson, Daniel M. Nover, Christopher M. Clark

**Affiliations:** 1 Tetra Tech, Inc., Research Triangle Park, North Carolina, United States of America; 2 Tetra Tech, Inc., Fairfax, Virginia, United States of America; 3 Office of Research and Development, U.S. Environmental Protection Agency, Washington, District of Columbia, United States of America; 4 School of Engineering, University of California–Merced, Merced, California, United States of America; Centro de Investigacion Cientifica y de Educacion Superior de Ensenada Division de Fisica Aplicada, MEXICO

## Abstract

A warming climate increases thermal inputs to lakes with potential implications for water quality and aquatic ecosystems. In a previous study, we used a dynamic water column temperature and mixing simulation model to simulate chronic (7-day average) maximum temperatures under a range of potential future climate projections at selected sites representative of different U.S. regions. Here, to extend results to lakes where dynamic models have not been developed, we apply a novel machine learning approach that uses Gaussian Process regression to describe the model response surface as a function of simplified lake characteristics (depth, surface area, water clarity) and climate forcing (winter and summer air temperatures and potential evapotranspiration). We use this approach to extrapolate predictions from the simulation model to the statistical sample of U.S. lakes in the National Lakes Assessment (NLA) database. Results provide a national-scale scoping assessment of the potential thermal risk to lake water quality and ecosystems across the U.S. We suggest a small fraction of lakes will experience less risk of summer thermal stress events due to changes in stratification and mixing dynamics, but most will experience increases. The percentage of lakes in the NLA with simulated 7-day average maximum water temperatures in excess of 30°C is projected to increase from less than 2% to approximately 22% by the end of the 21^st^ century, which could significantly reduce the number of lakes that can support cold water fisheries. Site-specific analysis of the full range of factors that influence thermal profiles in individual lakes is needed to develop appropriate adaptation strategies.

## Introduction

A warming climate is expected to result in increased lake water temperatures, presenting a threat to lake water quality and ecosystem health [1]. Surface water temperatures are closely tied to atmospheric forcing due to ready exchanges between the water surface and air. In a recent review, [2] found an average global rate of warming of lake surface waters of 0.34°C per decade between 1985 and 2009. Surface warming rates are highly heterogeneous, and depend on interactions among climatic and lake morphometric factors. The greatest increases occurred in seasonally ice-covered lakes in areas where temperature and solar radiation are increasing while cloud cover is decreasing (0.72°C per decade [2]).

Warming of surface waters also increases the strength of thermal stratification [3], leading to reduced mixing and, in some deeper lakes, a decrease in summer temperatures at depth [4, 5]. Such changes have direct implications for lake ecology. A review by [6] suggests that climate change has already altered abundance, growth, and recruitment of some North American inland fish populations, with particularly strong effects noted on cold water species. Changes in phenology and range are noted, resulting in altered structure and function of fish assemblages. The physiological basis of climate change effects on fish is reviewed by [7], who note the importance of aerobic scope (the difference between maximum and standard metabolic rate) in determining a species functional thermal tolerance. Risk to species survival thus depends on exposure to annual maximum water temperature for a critical duration. Hypoxia can interact with high temperatures to reduce the functional thermal tolerance window [8, 9].

In a stratified lake, the epilimnion is mixed by wind, yielding low vertical gradients, although a secondary thermocline often develops during the afternoon when wind mixing is low, resulting in less extreme temperature maxima near the thermocline. Trends in the temperature regime of lake mid-level and bottom waters are less well documented than changes at the surface. In deeper waters, temperature also depends strongly on mixing and stratification processes, which can also be expected to change under an altered climate. A more complete understanding of potential changes in lake thermal dynamics, including deeper waters, has important implications for anticipating and managing the effects of climate change on water quality and aquatic ecosystems.

The hypolimnion of a stratified lake generally maintains cooler temperatures; however, the hypolimnion may not be available as a refuge from thermal stress if dissolved oxygen is depleted, as is often the case in eutrophic lakes. We therefore focus on changes in maximum water temperature just above the thermocline, which approximates the interface between waters above with adequate dissolved oxygen replenishment (by reaeration and mixing) and waters below with cooler temperatures that provides a refuge in which sensitive species are more likely to survive extreme thermal events.

Our previous work [10] evaluated lake thermal and mixing response to changing climate in the mid-21^st^ century using the one-dimensional (vertical) Lake, Ice, Snow, and Sediment Simulator (LISSS) model [11]. LISSS simulations use lake geometry (e.g., surface area, depth) and climate forcing including air temperature, pressure, precipitation, humidity, wind, shortwave radiation, and downward longwave radiation to predict time series of temperature and mixing dynamics throughout the water column, including the temporal evolution and position of the thermocline. The study did not simulate actual lakes. Rather, [10] evaluated the response of 27 lake “archetypes” (a 3 x 3 x 3 matrix of depth, surface area, and water clarity characteristics) in 11 baseline hydroclimatic settings (representative of different geographic locations in the U.S.) to a range of mid-21^st^ century climate change scenarios. The analysis was informative as to how the physical characteristics of lakes affect temperature sensitivity to climate change, but did not inform upon how climate change effects could potentially be distributed in actual lakes throughout the contiguous U.S. (CONUS).

This study demonstrates a method to extrapolate from detailed physics-based models to a national scale screening assessment using only simplified and readily available inputs (e.g., monthly air temperature projections). We accomplish this through use of a surrogate model that approximates the response surface based on a “training” set of dynamic simulation model output. We combine the methods of [10] with the surrogate model to develop a sensitivity analysis of the potential risk of climate change-induced water temperature extremes for lakes of the CONUS included in the National Lakes Assessment (NLA) database [12]). Surrogate models have been developed and widely used in information theory [e.g., 13, 14]. Recent applications in water resources are summarized in [15]. Most applications in the water sector have been for model calibration purposes; however, the method is equally applicable to extension of modeling results to a broader population for which the original, detailed model has not been run.

## Methods and data

### National lakes assessment database

The 2007 NLA database is a statistical sample of lakes throughout the U.S. The NLA is part of EPA’s National Aquatic Resource Surveys program, which aims to provide statistically valid data describing water resource quality conditions across the country. The NLA provides information on over 1,000 lakes in the CONUS, collected using consistent methodology, as a means to summarize the state of the nearly 50,000 natural and man-made lakes that are greater than 10 acres (0.04 km^2^) in area and over one meter deep. As such, the NLA provides an ideal basis for broad-scale inference about the future status of U.S. lakes as potentially affected by climate change. Specifically, we use the NLA coupled with the surrogate model to extend the results of LISSS simulations to actual lakes across the CONUS. In this analysis, we removed lakes with incomplete morphometric data and filtered the sample to include lakes with 0.1 to 100 km^2^ surface area and 2 to 30 m depth (consistent with the training data set described below), resulting in a total of 898 lakes.

### Surrogate model

#### Training data set

Developing a surrogate model of the response surface suitable for application to the NLA dataset requires a broad sampling of the model domain. To accomplish this, we used a training data set of 1,701 LISSS model simulations using climate forcing series developed previously by Butcher et al. 2015 (simulations for 9 climate time series locations x 7 climate scenarios x 27 versions of morphometric and water quality conditions).

Meteorological forcing required for LISSS consists of hourly series of air temperature, pressure, precipitation, humidity, wind speed, shortwave radiation, and downward longwave radiation. Baseline hydroclimatic conditions in the training data set include nine first-order weather stations located in different U.S. regions ranging from humid sub-tropical Tampa, FL (mean temperature 22.3°C) to cold, high-elevation Sugarloaf Reservoir, CO (mean temperature 4.6°C). See S1 Text, S1 Fig, S2 Fig, and S1 Table in the supporting information for additional details. The meteorological time series (one historic and six future climate series at each location) used by [10] in the training data set are a subset of those developed for a U.S. EPA study of potential climate effects on hydrology and water quality in large U.S. watersheds [16, 17]. Historical time series cover the period 1971–2000. Climate change scenarios are derived from six high-resolution simulations archived by the North American Regional Climate Change Assessment Program (NARCCAP; [18]) for 2041–2070. The NARCCAP simulations use regional climate models (RCMs) to dynamically downscale output from Global Climate Models (GCMs) used in the Intergovernmental Panel on Climate Change (IPCC) 4th Assessment Report [19] to a 50x50 km^2^ grid over North America and provide a full suite of physically consistent meteorological variables. The climate change scenarios used by [10] in the training data set, while not comprehensive of all potential climate futures, represent a plausible range of potential mid-century changes appropriate for training the surrogate model.

Each meteorological forcing scenario (one historic and six future climate series at each hydroclimatic location) was combined in 27 replicates with a quasi-random sampling realization of three key lake characteristics: maximum depth, surface area (or average fetch), and water transparency (extinction coefficient). The lake characteristics are spread out to maximize coverage of the potential range of interest, but are not truly randomized because true random sampling paradoxically results in inefficient clumping of scenario conditions; rather they are based on a quasi-random scheme. Specifically, we use the quasi-random space-filling sequence of 0–1 variables proposed by [20] and refined by [21; see also 22] to derive the lake characteristics distribution for training. In a Sobol sequence, successive sample points are located to fill gaps in the previously generated distribution, maximizing coverage of the response surface sample space (see S3 Fig). Model code and results of the training runs are available in an online repository (DOI 10.17605/OSF.IO/4r44z).

All future climate change scenarios for all hydroclimatic locations in the training data set show an increase in average annual and seasonal air temperatures. Not surprisingly, this results in an increase in the predicted average summer surface water temperature and total heat content of the water column of the simulated lakes. LISSS model results for maximum temperatures above the thermocline (operationally defined for each simulation day as the layer of the water column deeper than 1 m and immediately above the point of maximum change in the vertical temperature profile during stratification or the bottom water layer during mixed conditions) are more complex, particularly as regards annual maxima.

Many lacustrine species are constrained by thermal tolerances during episodic high temperature events. Increases in temperature are a particular concern for fish. Since the 1970s [23, 24], U.S. EPA has recommended evaluating chronic risks to fish from elevated temperature in terms of MWAT–the maximum weekly average temperature, calculated as the highest annual average of seven consecutive daily maxima (also referred to as the 7DADMax temperature). MWAT tolerances have been developed for both streams and lakes (e.g., [25]). The majority of lake simulations in the training data set have a projected increase in MWAT above the thermocline; however, there are also a number of cases (slightly under 9%) that have a projected decrease under future climate conditions due to changes in timing and depth of stratification. In this study, we use MWAT as a summary index of thermal conditions in lakes, and use this index to suggest potential risk to sensitive fish species.

#### Response surface surrogate model development

The surrogate model for MWAT is based on Gaussian Process regression. In Gaussian Process regression, the response surface is simulated as the sum of a potentially infinite set of Gaussian (i.e., normally distributed) variables subject to a covariance structure that can be specified based on a regression against external variables (e.g., lake morphometry and climate conditions). Once established, the response surface surrogate model can be applied to any set of lake characteristics and climate projections that are reasonably encompassed by the domain of the training data set.

Gaussian Processes have seen extensive recent development for supervised machine learning problems, where a surrogate is required for an intrinsically complex process and the problem is one of learning input-output mappings from empirical data [26]. Gaussian Processes take advantage of the fact that the sum of normally distributed (Gaussian) random variables is also Gaussian and even an infinite set is tractable. If the variable of interest is normalized so that it has a mean of zero, the resulting distribution also has a mean of zero and the prediction of individual points depends only on the covariance structure. The covariance structure that provides the best fit across all training points is the posterior distribution that combines model and observations. The Gaussian Processes approach is shown to be consistent with a one-level ANN model, kriging approaches, and a Bayesian interpretation [26]. The Bayesian perspective leads directly to a description of the conditional probability distribution of a prediction at any point conditioned on the training data. For regression problems, if the dispersion about the regression line is assumed to be Gaussian (regardless of the complexity of the regression relationship itself) the analytical problem is tractable and efficient, being governed by inversion of an *n* x *n* matrix for a problem with *n* training data points. In this paper, we implement Gaussian Process regression using the pyGPs Python package [27] using MWAT above the thermocline as the response variable. Code and output are provided in the online data repository (DOI 10.17605/OSF.IO/4r44z).

**Fig 1:**
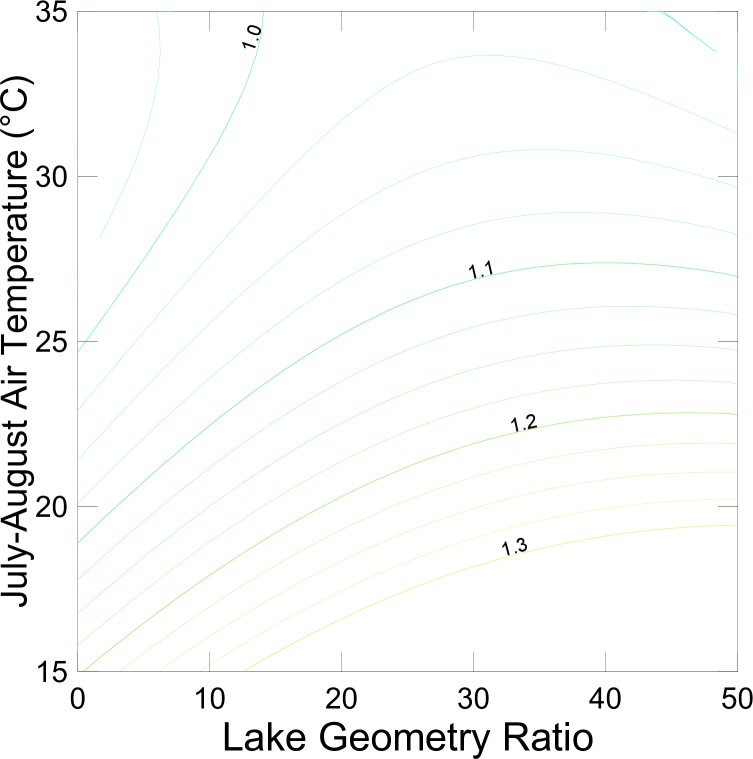
Change in MWAT above the thermocline as a function of July-August air temperature and lake geometry ratio in the training data (with quadratic smoother applied).

The relationship between simulated MWAT and summer air temperature in the training data set is complex and dependent on site-specific climatic conditions. The likelihood of strong stratification in a lake corresponds to a low lake geometry ratio (Area^0.25^/H_max_; units L^-0.5^), where H_max_ is the maximum depth [28]. Sites with larger relative changes in MWAT tend to be exhibit lower historic summer air temperatures and higher geometry ratios, representing lakes with weaker stratification (**Fig 1**). The smaller response at higher summer air temperatures is likely due in part to increased evaporative cooling. Lakes in which LISSS projects a reduction in future MWAT tend to be both small and deep with a geometry ratio less than 10.

To fit the surrogate model, we first normalized the training data set to a zero mean process, as *Y*_i_ = (*X*_i_—$\bar{X}$/*σ*), where *X*_i_ represents the MWAT estimate from a model run, $\bar{X}$ is the mean value of all *X*_i_ (25.65°C) and *σ* is the standard deviation of the training *X*_i_ (3.265). The Gaussian Process regression analysis of the model response surface was conducted in a number of parametric forms (linear and non-linear) informed by exploratory sensitivity analyses conducted on the training data set. The final surrogate model representation was selected based on Akaike Information Criterion (AIC; [29]) scores relative to the detailed model results. We evaluated reliability of the surrogate model through bootstrap cross-validation and comparison to an independent machine learning approach (Random Forest regression).

### Climate change scenarios–NLA lakes

The climate change scenarios used in our assessment of the potential climate change effects on NLA lakes are different from those of the training dataset. In the NLA assessment, we consider a larger ensemble range of scenarios based on modeling results from the Coupled Model Intercomparison Project Phase 5 (CMIP5) associated with the fifth assessment report of the IPCC [30], and include results through the end of the 20^th^ century. Scenarios are based on the Multivariate Adaptive Constructed Analogs (MACA) statistically downscaled data (to a 4 km x 4 km scale). MACA includes output from a large number of CMIP5 experiments (30+ GCMs under multiple representative concentration pathways (RCPs) of greenhouse gas emissions). The MACA method [31] has two advantages that make it preferable to other statistical downscaling methods for simulating local waterbody responses: (1) it provides simultaneous downscaling of precipitation, temperature, humidity, wind, and radiation (rather than just precipitation and temperature), providing physical consistency in the energy balance, and (2) the method uses a historical library of observations (analogs) to construct the downscaling such that future climate projections are distributed from the monthly to the daily scale in comparison to months that exhibit similar characteristics in the historical record. MACA data were accessed through a THREDDS server (http://maca.northwestknowledge.net/) that allowed results to be extracted for each lake location.

To characterize the range of climate risk we screened available MACA scenarios for output of six GCMs under the RCP 8.5 pathway preferring candidates that (1) are evaluated as having good skill on prediction of warm-dry conditions (i.e., conditions under which maximum lake thermal increase is expected) in temperate latitudes [32], (2) have high spatial resolution [33], and (3) exhibit a range of projected increases in annual mean maximum air temperatures across the Continental U.S. based on the USGS National Climate Change Viewer (NCCV; [34]). The six selected model runs under RCP 8.5 are CNRM-CM5, HadGEM2-CC365, IPSL-CM5A-MR, BCC-CSM1-1, GFDL-ESM2M, and MRI-CGCM3. The NCCV shows a range of increase in annual mean maximum temperature (2075–2099 vs. 1950–2005) across the CONUS of 3.1°C (MRI-CGCM3) to 7.1°C (HadGEM2-CC365) for these GCM runs.

The MACA daily output was downloaded for each NLA location for maximum and minimum air temperature, specific humidity, precipitation, downward shortwave radiation, and near surface wind. The energy variables were used to calculate potential evapotranspiration (PET) using the FAO56 implementation of the Penman-Monteith energy balance method [35].

## Results

### Surrogate model performance

The surrogate model uses as input only readily available climate variables such as monthly average air temperature. Analysis of simulations in the training data set in [10] suggested that MWAT above the thermocline was correlated with the average July-August air temperature, the January air temperature (indicative of the ice regime), PET, light extinction/water clarity, and lake geometry (depth, surface area) (see S3 Table). Exploratory multivariate regression models provided reasonable results for specific depth ranges, but appeared to depend on depth in a non-linear fashion, suggesting the need for a more sophisticated analysis. For surrogate model development, water clarity is represented by the light extinction coefficient (m^-1^) that controls the depth of light penetration. Lake geometry is summarized by the geometry ratio [28], which [36] found to be a useful discriminator of potential lake mixing behavior. Evaporation affects lake temperature directly, but PET is likely also important as a surrogate for changes in wind and incident solar radiation.

We investigated surrogate model fit across the selected set of explanatory variables and a range of covariance kernels provided by pyGPs [27]. Best fit based on AIC was provided by the squared exponential plus rational quadratic kernel using all explanatory variables. Further details of the Gaussian Process model fit are provided in the supporting information (S2 Text, S4 Table, and S5 Table). Bootstrap cross-validation and comparison to Random Forest regression (S2 Text) confirm the reliability of the surrogate model representation of LISSS simulation results. In the bootstrap tests, average error (for untransformed predictions) was -0.0042°C and average absolute error was 0.588°C–suggesting that the model fit is unbiased but somewhat imprecise.

The supplemental information provided with this article provides additional details on the surrogate model fit. Predictions using Gaussian Process regression are a linear combination of the points in the training data set with weights specified by the maximum likelihood estimate of the covariance matrix that are based on distances in each predictor metric. The surrogate model does not predict point estimates directly from the independent variables, only the covariance structure.

**Fig 2:**
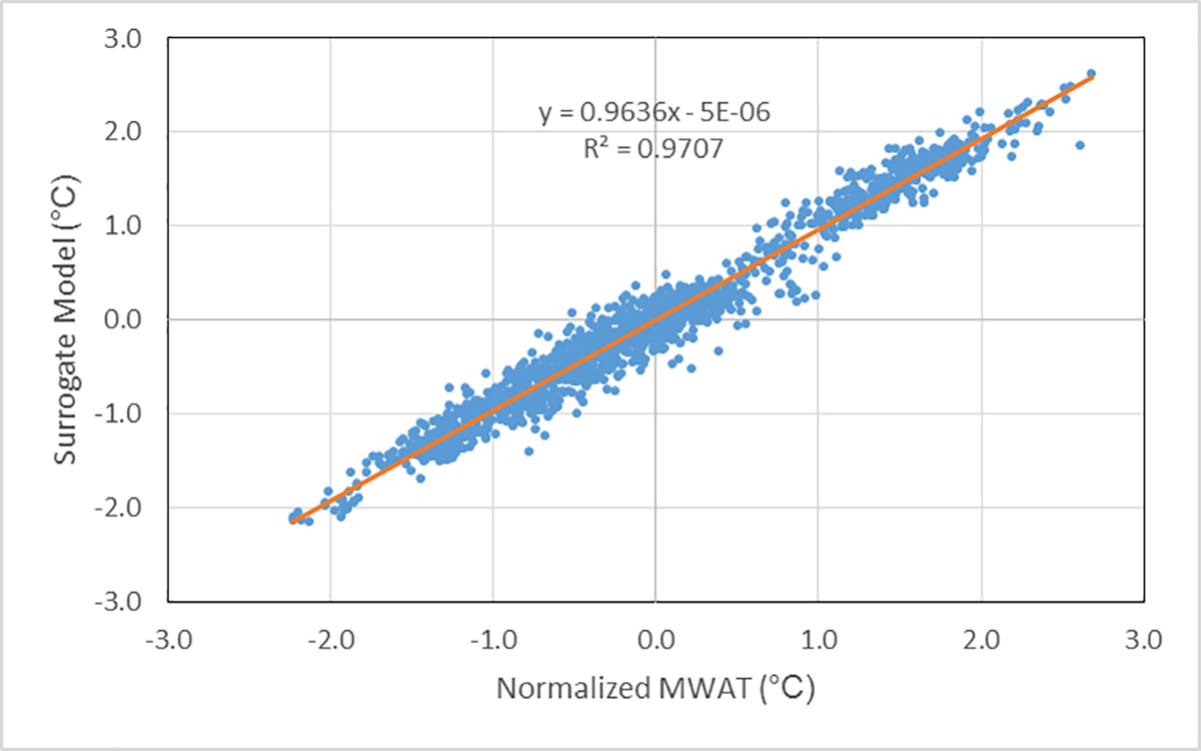
Surrogate model fit to LISSS model runs for MWAT above the thermocline.

The surrogate model omits, by design, many factors that could influence lake responses to climate change to reduce the predictive variables to a set that is commonly available for lakes in the NLA. This simplified model is still a credible predictor, explaining 97% of the variability in the training data set with a root mean squared error of 0.54°C on de-normalized results and an average absolute error of 0.41°C (**Fig 2**).

To establish the credibility of the approach, surrogate model performance for MWAT was evaluated against publicly available, long-term monitoring data for five lakes (Castle Lake, CA [37]; Lake Mendota, WI [38]; Crystal Lake, WI [38]; Sparkling Lake, WI [38], and Lake Lacawac, PA [39]). Over a combined total of 116 observation years, the surrogate model had an average relative error on MWAT of 1.82% (0.28°C) and an average relative absolute error of 7.23% (1.59°C), both well within the 95% confidence limits of the Gaussian Processes surrogate model (approximately ±20%). It did appear, however, that the Gaussian Processes model was biased high for Lake Mendota (+12%) and biased low for Lake Lacawac (-8%), emphasizing the need for site-specific studies to account for conditions applicable to individual lakes.

### Application of surrogate model to NLA lakes

The NLA is a statistical sample, not an exhaustive catalog of lakes in the CONUS. As such, it provides a good basis for evaluating the broad scale range of climate change effects on lake thermal structure and mixing in different regions of the CONUS. Results for individual lakes may differ based on local conditions. Importantly, it should also be noted that results are only a benchmark of potential climate change effects and do not include other potential effects that may be associated with future changes such as increased pollutant loading and changes in water clarity.

#### Variability among NLA lakes

We first present results obtained from application of the surrogate model using the median monthly air temperature and PET series across the six climate change scenarios, which suggest a range of potential future changes. We later evaluate and discuss the range of potential outcomes across the ensemble of climate change scenarios (e.g., different GCMs).

**Fig 3:**
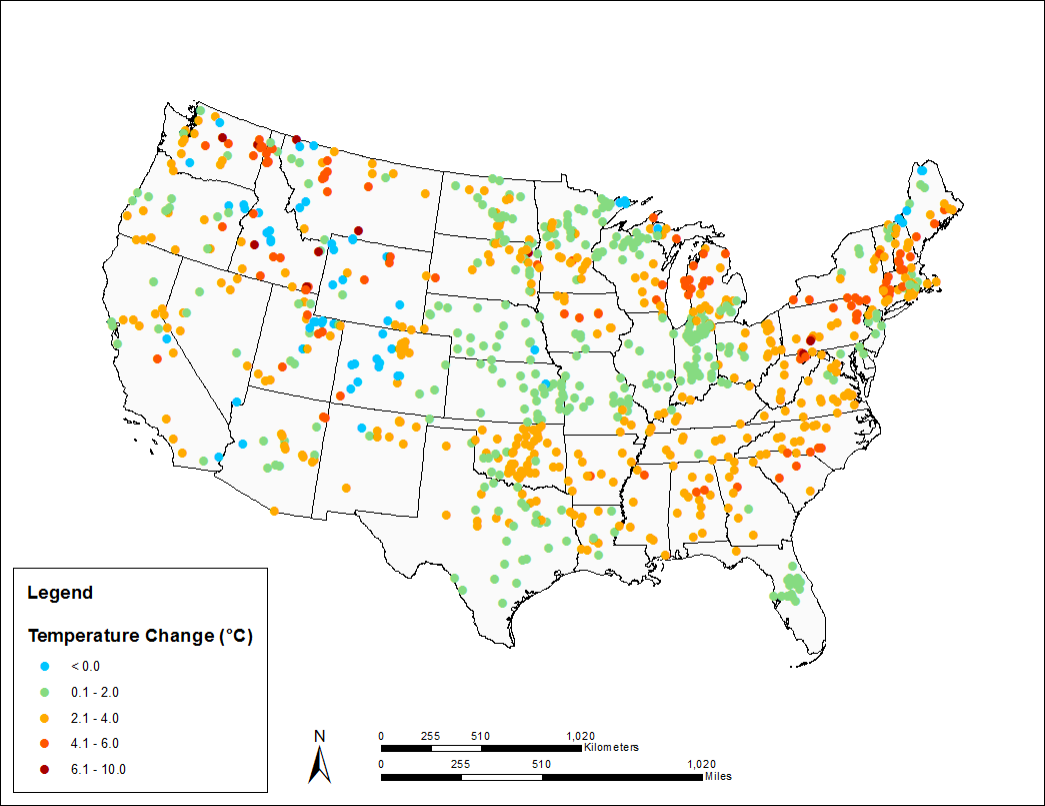
Simulated changes in summer MWATs above the thermocline for NLA lakes, ca. 2065 (median across climate scenarios).

**Fig 4:**
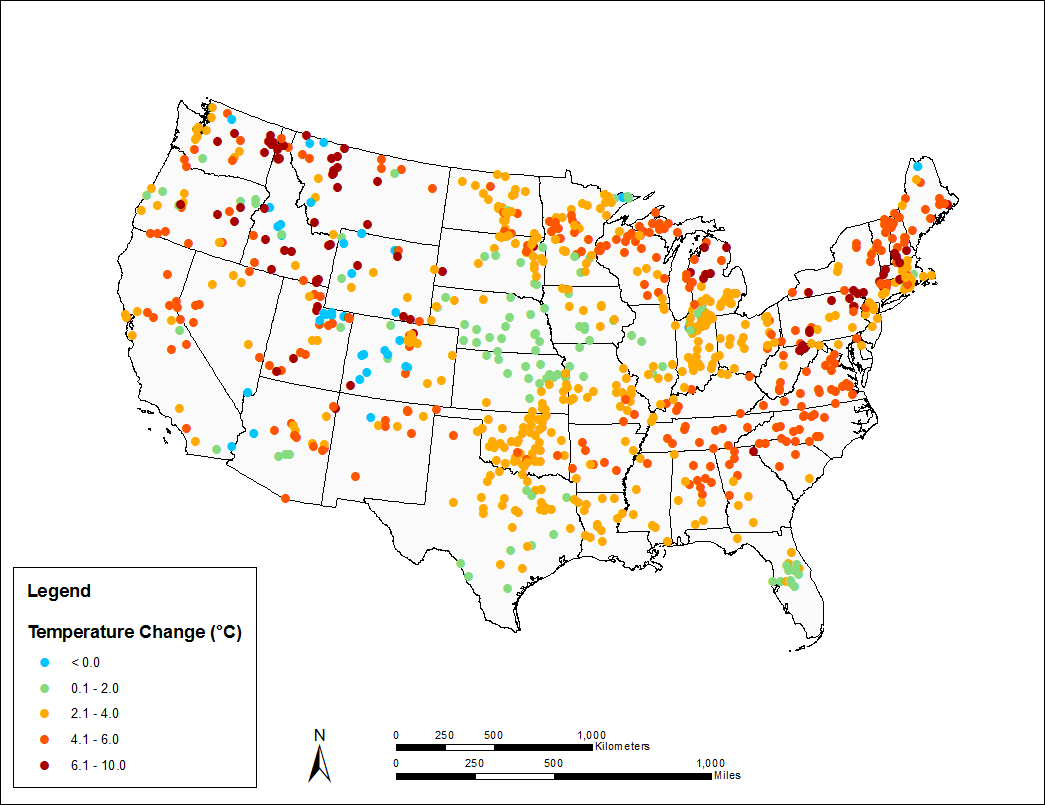
Simulated changes in summer MWATs above the thermocline for NLA lakes, ca. 2085 (median across climate scenarios).

Changes in summer MWAT, by lake, across all climate scenarios, are summarized geographically in **Fig 3**, for the 30-year time slice centered on 2065, and **Fig 4**, for the 30-year time slice centered on 2085. The average summer MWAT across all NLA lakes increases from 24 to 26.2°C by mid-century (ca. 2065) and to 27.5°C by late-century (ca. 2085). Towards the end of the century the maximum projected change for individual lakes is as great as 9.5°C, while the average projected change is an increase of 3.5°C. Lakes with the greatest absolute changes in MWAT tend to have low geometry ratios associated with small surface area and relatively cold historic baseline temperatures. Consistent with the training data set, a small percentage of the lakes (3% mid-century, 2% late-century) have a projected decline in the MWAT. These are primarily (but not exclusively) lakes with low geometry ratios at higher elevations in the Rockies or near the Canadian border. The overall geographic patterns are complex, reflecting the interaction of the heat budget and strength and timing of stratification in individual lakes.

**Fig 5:**
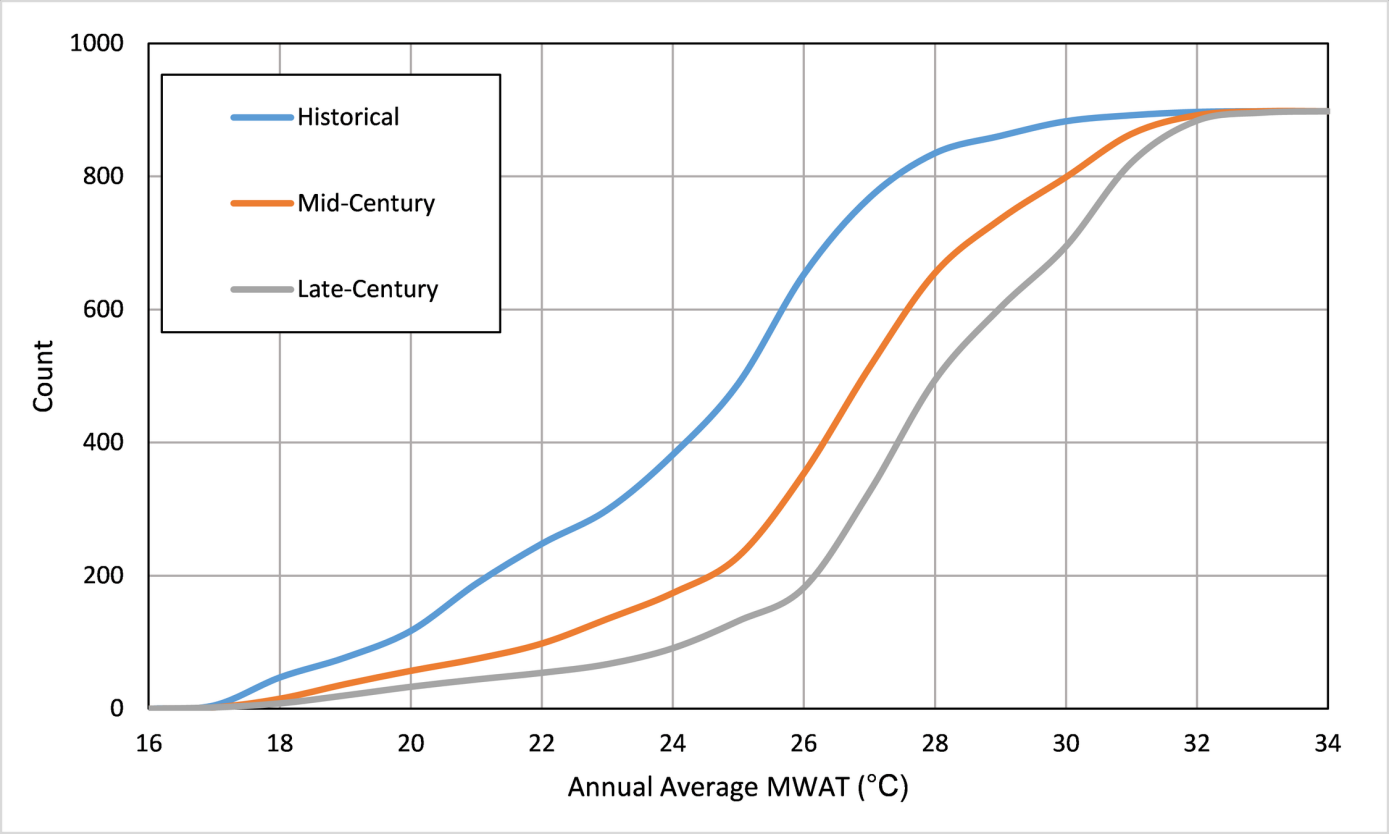
Cumulative frequency distribution of MWAT.

The cumulative frequency distribution of MWATs predicted by the surrogate model for the 898 NLA lakes included in the study is shown in **Fig 5**. The percentage of lakes with MWAT exceeding 30°C (an approximate threshold for adverse effects on cold water fisheries) increases from 1.7% under recent historical climate to 11.1% by mid-century and 22.6% by late-century.

**Fig 6:**
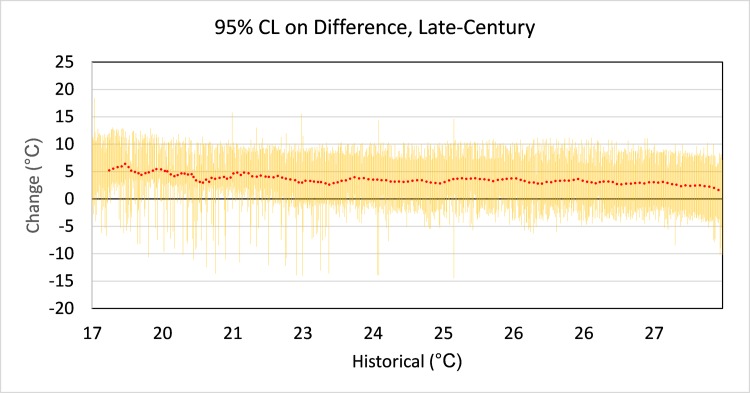
95% confidence limits on change in MWAT above the thermocline for NLA lakes, ca. 2085 (confidence limits and 25-point moving average on mean).

The surrogate model estimates are a statistical approximation of the LISSS model results and thus subject to uncertainties associated with specific physical characteristics resolved by LISSS simulations. The 95% confidence limits on the mean projections from the surrogate model (**Fig 6**) show a relatively wide range of uncertainty associated with the surrogate model projections for many lakes, even independent of the additional variability associated with individual climate scenarios. It does appear, however, that there is a risk of greater temperature increases in lakes that currently have colder MWATs. The causes underlying this relationship are many and complex; however, at the colder end of the spectrum reductions in the extent of ice cover facilitate greater total warming, while at the warmer end of the spectrum increases in thermal stability and earlier onset of stratification can preserve cooler temperatures below the depth of light penetration.

### Variability among climate scenarios

Simulations for each NLA lake show a range of potential changes across the ensemble of climate change scenarios (GCMs) evaluated, with average predicted gains in MWAT above the thermocline by GCM ranging from 2.6 to 4.5°C. For the six sampled GCMs, the rank order from smallest to largest average gain for mid-century is GFDL-ESM2M < MRI-CGCM3 < CNRM-CM5 < BCC-CSM1-1 < IPSL-CM5A-MR < HadGEM2-CC365, and for late century, MRI-CGCM3 < GFDL-ESM2M < CNRM-CM5 < BCC-CSM1-1 < IPSL-CM5A-MR < HadGEM2-CC365, consistent with the GCM-predicted changes in average annual air temperature across the CONUS. One-way ANOVA on GCM confirms that the means are significantly heterogeneous (F_5, 5, 382_ = 147.4, p < 0.001 for late century), with HADGEM2-CC365 and IPSL-CM5A-MR predicting significantly larger increases than the other GCMs. 88% of the variance late-century is associated with GCM.

The magnitude of predicted change also varies spatially. By EPA region, the projected late-century increases are significantly greater (Tukey-Kramer test, p <0.05) for Regions 1, 2, 3, 4, 9, and 10 (predominantly coastal) compared to Regions 5, 6, 7, 8 (predominantly continental). This spatial variability likely reflects a variety of competing factors in the complex response of MWAT, which involves the interaction of heat exchanges, stratification depth and stability, and ice regime. In general, smaller changes in MWAT appear to be associated with higher elevation, higher existing MWAT, lower geometry ratio, and lower water clarity.

## Discussion and conclusions

A warming climate will increase thermal inputs to lakes, increasing average water temperatures. Lake thermal response to climate change is complex because it involves alteration to both total heat content and stratification regime. Stratification responses to changing climate have been observed worldwide and are primarily controlled by lake morphometry and existing temperature baseline [40], consistent with our finding. These changes have a wide range of implications for lake water quality and ecosystems.

This study demonstrates use of a Gaussian Process surrogate model to extend results of dynamic simulation to a national population of lakes based only on readily available inputs. The method performs well, although it is limited by the assumptions underlying the 1-D dynamic model and the uncertainty associated with prediction from a simplified set of explanatory variables. We apply the surrogate model to develop a national-scale sensitivity analysis of the risk of climate change-induced responses of water temperature in lakes across the CONUS that are included in the NLA. Results suggest the range of effects that may be encountered, their regional distribution, and their dependence on lake physical characteristics and climate forcing–but are not intended as quantitative predictions for individual lakes.

The national sensitivity analysis focuses on one aspect of lake temperature–thermal stress within the epilimnion as indexed by MWAT above the thermocline. This metric is a significant factor for determining the functional thermal tolerance window for fish, especially for cold water species such as trout and salmon. During periods of high thermal stress in stratified lakes fish survive in cooler waters at the bottom of the mixed zone that is in contact with the atmosphere [41], which provides a refuge of last resort where both thermal/metabolic and aerobic requirements can be met. It has been argued [42] that rising temperatures will likely “tighten a metabolic constraint” on marine ecosystems. The results of our analysis suggest there is a similar risk of reduction in the extent of zones favorable to survival of many fish species in freshwater lakes, consistent with the review by [9].

Analysis of temperate freshwater lakes is complicated by seasonal stratification and the presence of winter ice cover, both of which affect the seasonal position and extent of zones in which species can survive periods of high thermal stress. Lake ecosystems are particularly vulnerable to climate change because many species within these ecosystems have limited abilities to disperse to lakes with more favorable conditions [43]. Success of a species in a stratified lake is potentially constrained by the frequency of occurrence of events of high thermal stress in the epilimnion, especially if oxygen is depleted below the thermocline.

Critical thermal maxima for cold water fish species such as salmonids are typically cited as in the range of 29–32°C (e.g., [25]). The MWAT above the thermocline results from the interaction of factors that control heating of the water column, strength of stratification, and the depth of the thermocline in lakes. More turbid lakes can have lower thermocline MWATs because light energy does not penetrate as deeply. In clearer lakes, the critical temperature at the bottom of the mixed zone depends on the interaction between light penetration and the depth of the thermocline during thermal maximum events.

Our analysis suggests that lakes in the CONUS may, on average, experience an increase in MWAT of about 3.5°C by the end of the century, which is sufficient to increase the average annual maximum from well below 30°C to values in the 31–32°C range for many lakes. If realized, this presents a risk of substantial reductions in the number of lakes in which populations of cold water species will be viable if hypolimnetic hypoxia occurs. This conclusion does not apply to stratified lakes in which the cooler hypolimnion remains oxygenated during extreme thermal events. Presence of an oxic hypolimnion is correlated to chlorophyll *a* concentration, strength of stratification, elevation, Secchi depth, and lake geometry [44].

The model-based findings presented here are in general consistent with a recent summary analysis of lake temperature and stratification trends in northeastern North America [45], which, for the first time, assembles available observations on thermal profiles throughout a region with many intensively studied lakes. The authors confirm that surface temperatures and thermal stratification strength have generally increased over the last 40 years, while deeper water temperatures show more complex responses. For deeper water, some lakes exhibited warming and others cooling. Secchi depth and the interaction of Secchi depth with lake depth were important explanatory variables for response of individual lakes, as were weather conditions during spring mixing. Geographic patterns reported in [45] are complex, although lakes nearer to the coast were more likely to exhibit cooling of deep waters.

Our study is based on a 1-D lake model that does not incorporate multi-dimensional processes such as internal waves and mixing induced by lateral inflows; it also does not address the many other changes in lake conditions and watershed forcing that may occur in response to climate change. The direct thermal response of lakes to altered climate can be affected by many factors, including changes in amount, timing, and temperature of surface and groundwater inflows, reductions in summer lake levels, and potential changes in water clarity that affect the vertical distribution of heat. For some lakes, climate change may also result in increased watershed loads of nutrients and sediment [16]. Increases in strength of stratification and thermal stability of the water column may increase rates of hypolimnetic oxygen depletion, which in turn could facilitate recycling of nutrients from lake sediments, while altered timing of fall overturn could change how and when hypolimnetic nutrients are mixed into the epilimnion. The planktonic and benthic communities at the base of the aquatic food web may also change in ways that affect fish populations as well as other ecosystem services provided by lakes. For instance, increased water temperature accompanied by potential changes in ice out timing and mixing regimes may result in shifts toward more turbid, plankton dominated systems [46, 47, 48]. Of particular concern, higher temperatures in combination with nutrient enrichment are shown to increase the risk of harmful algal blooms and the proliferation of toxin producing cyanobacteria [49, 50], which could impair both biotic and human uses of lakes.

The results we present are obtained with a simplified surrogate model that is appropriate for national scoping, but is not sufficient to fully describe responses in real, three-dimensional lakes. Additional investigations using process-based (rather than surrogate) models would help elucidate apparent spatial patterns that may reflect ecoregional characteristics.

Development of strategies for responding to climate change will need to take account of the full range of climate-related stressors and site-specific studies will be needed for individual lakes where valued resources appear to be at risk. Responding to thermal aspects of climate change may require development of interventions, such as manipulation of the vertical distribution of lake outflows that alter the mixing regime. Adaptation to the full range of climate-associated stressors in lakes will require holistic management of lakes and their watersheds.

## Supporting information

S1 Text Constructing the training data set. (DOCX)

S2 Text Gaussian processes regression: Model fit and reliability testing. (DOCX)

S1 Fig Location of meteorological stations used in training data set. (DOCX)

S2 Fig Distribution of average annual precipitation and temperature (1971–2000) at meteorological stations used in training data set. (DOCX)

S3 Fig Example quasi-random sobol sequence of 0–1 triplets used to create the LISSS training dataset. (DOCX)

S1 Table Identification of meteorological stations used in LISSS training dataset. (DOCX)

S2 Table GCMs and RCMs for NARCCAP climate scenarios. (DOCX)

S3 Table Correlation coefficients for the simulations of MWAT above the thermocline in Butcher et al. [2015]. (DOCX)

S4 Table AIC obtained from optimizing seven covariance kernel functions to training data. (DOCX)

S5 Table Optimized posterior hyper-parameters for selected covariance model (sum of rational quadratic kernel and squared exponential kernel). (DOCX)
