# Supplementary material for: Estimating future temperature maxima in lakes across the United States using a surrogate modeling approach
Source: PLoS One. 2017 Nov 9;12(11):e0183499. doi: 10.1371/journal.pone.0183499 (PMC5679518; doi:10.1371/journal.pone.0183499)
Supplement: S1 Text — (DOCX) [file pone.0183499.s001.docx]

Supporting Information for

Estimates of Future Temperature Maxima in Lakes across the United States using a Surrogate Modeling Approach

Jonathan B. Butcher^1^, Tan Zi^2^, Michelle Schmidt^1^, Thomas E. Johnson^3^, Daniel M Nover^4^, and Christopher M. Clark^3^

^1^Tetra Tech, Inc., Research Triangle Park, NC; ^2^Tetra Tech, Inc., Fairfax, VA; ^3^ U.S. Environmental Protection Agency, Office of Research and Development, Washington, DC;
^4^ University of California – Merced, School of Engineering.

S1 Text. Constructing the Training Data Set

The training data set is based on existing (1970-2000) observed meteorological time series and future (mid-21^st^ century) time series based on modifying the existing time series with a change factor approach based on the differences between Regional Climate Model simulations for 1971-2000 and 2040 -2071. S1 Figure and S1 Table show the nine station locations used in this exercise. Figure S2 shows that these stations cover a broad range of average annual temperature and average annual precipitation.

The potential future climate scenario timeseries at each location are derived from analysis of six high-resolution climate simulations archived by the North American Regional Climate Change Assessment Program (NARCCAP; [*Mearns et al.*, 2009]), as summarized in S2 Table. Full details regarding the creation of these time series may be found in *U.S. EPA* [2013] and in the supplemental material to *Butcher et al.* [2015].

Lake characteristics (for depth, surface area, and water transparency) for the training data set are assigned with a quasi-random space-filling Sobol sequence, as discussed in the main text. S3 Figure provides an example of the distribution of these points in three dimensions.

References

Mearns L.O., W.J. Gutowski, R. Jones, L.Y. Leung, S. McGinnis, A.M.B. Nunes, and Y. Qian, (2009), A regional climate change assessment program for North America, Eos Trans. AGU, 90: 311-312, doi:10.1029/2009EO360002.

U.S. EPA (Environmental Protection Agency), (2013), Watershed Modeling to Assess the Sensitivity of Streamflow, Nutrients, and Sediment Loading to Potential Climate Change and Urban Development in 20 U.S. Watersheds. EPA/600/R12/058F. National Center for Environmental Assessment, Office of Research and Development. <http://cfpub.epa.gov/ncea/global/recordisplay.cfm?deid=256912>.
