# Supplementary material for: Estimating future temperature maxima in lakes across the United States using a surrogate modeling approach
Source: PLoS One. 2017 Nov 9;12(11):e0183499. doi: 10.1371/journal.pone.0183499 (PMC5679518; doi:10.1371/journal.pone.0183499)
Supplement: S2 Text — (DOCX) [file pone.0183499.s002.docx]

Supporting Information for

Estimates of Future Temperature Maxima in Lakes across the United States using a Surrogate Modeling Approach

Jonathan B. Butcher^1^, Tan Zi^2^, Michelle Schmidt^1^, Thomas E. Johnson^3^, Daniel M Nover^4^, and Christopher M. Clark^3^

^1^Tetra Tech, Inc., Research Triangle Park, NC; ^2^Tetra Tech, Inc., Fairfax, VA; ^3^ U.S. Environmental Protection Agency, Office of Research and Development, Washington, DC;
^4^ University of California – Merced, School of Engineering.

S2 Text. Gaussian Processes Regression: Model Fit and Reliability Testing

Initial predictor variables for the Gaussian Processes regression were selected based on the correlations to MWAT above the thermocline in the simulation data set reported in Butcher et al. [2015}, shown in S3 Table. Gaussian Processes regression was implemented using the pyGPs Python package [*Neumann et al*., 2015]. The raw MWAT variables (*y*) are normalized to a zero mean process as $= \frac{\left( y- \bar{y} \right)}{\sigma}$ , where $\bar{y}$ is the mean of the observations and σ is the sample standard deviation. Potential explanatory variables considered are the average July-August air temperature (JulAugATEM, F), average January air temperature (JanATEM, F), Penman-Monteith potential evapotranspiration (in/mo), light extinction coefficient (Eta, m^-1^), and the lake geometry ratio of *Gorham and Boyce* [1989]. Eight covariance kernels were tested and optimized using the conjugate gradient approach. The covariance kernels are defined on the multidimensional separation of points *x* and *x’* as *k(x, x’)* as follows:

1. Squared exponential kernel: $k\left( x, x' \right)= \sigma^{2} exp\left( - \frac{{|\left| x-x^{'} \right||}^{2}}{2 l^{2}} \right)$.
2. Linear kernel: $k\left( x, x' \right)= \sigma^{2} x^{T}x'$.
3. Polynomial kernel: $k\left( x, x' \right)= \sigma^{2} \left( x^{T}x^{'}+c \right)^{d}$.
4. Rational quadratic kernel: $k\left( x, x' \right)= \sigma^{2} \left( 1+ \frac{{|\left| x-x^{'} \right||}^{2}}{2 \alpha l^{2}} \right)^{-\alpha}$.
5. Sum of rational quadratic kernel and squared exponential kernel.
6. Matern kernel: $k\left( x, x' \right)= \sigma^{2} \frac{2^{1- \nu}}{\Gamma(\nu)} \left( \frac{\sqrt{2 \nu} |d|}{l} \right)^{\nu} K_{\nu}\left( \frac{\sqrt{2 \nu} |d|}{l} \right).$
7. Squared exponential kernel with unit magnitude: $k\left( x, x' \right)= exp\left( - \frac{{|\left| x-x^{'} \right||}^{2}}{2 l^{2}} \right)$.

Here *d = x – x’* and σ is the standard deviation of the noise fluctuation. The parameter *l* is the characteristic length-scale of the process, *K(ν)* is the modified Bessel function of order *ν*, *Γ(ν)* is the gamma function evaluated at *ν*, *α* is a shape parameter, and *c* is a constant.

AIC values from optimizing each kernel to the training data with all explanatory variables are shown in S4 Table. The minimum AIC is obtained with the sum of squared exponential and rational quadratic kernel (model 5). Calculation of AICs with all subsets of explanatory variables yields the minimum AIC when all variables are included. S5 Table provides the optimized posterior hyper-parameters for the selected covariance model.

The reliability of the Gaussian Processes regression representation of the training data was tested in two ways – first, through the application of Random Forest regression, a classification-based empirical approach entirely independent of the Gaussian Processes analysis, and second through bootstrap cross-validation experiments.

For the Random Forest regression we used the “randomForest” package (*Liaw and Wiener* [2002]) in R software (*R Core Team* [2017]). The raw (not transformed) predictors and response were used as model inputs. The response is a continuous variable.

Three Random Forest hyper-parameters were “tuned” or optimized for this model: (1) The number of predictors randomly sampled at each split (an integer value) was optimized via a grid search of values {1, 2, 3, 4}. A value of 2 was the best, based on out-of-bag (OOB) mean squared error (MSE) criterion. (2) The minimum leave size of each tree (an integer value) was optimized via a grid search of values {2, 5}. A minimum leaf size of 5 was the best, based on OOB MSE. (3) Finally, the number of trees within the random forest was selected to be 2,500, based on a visual inspection of a plot of OOB MSE vs number of trees.

Each tree within the random forest is composed of samples with replacement from the training dataset. Therefore, for a given tree, there are data that were not used to fit that tree’s model. These unused data are referred to as out-of-bag (OOB) samples and provide a test of model performance. The Random Forest regression yielded a similar fit to the Gaussian Processes model for MWAT, although the quality of fit for Gaussian Processes was slightly better (R^2^ and untransformed RMSE of 0.949 and 0.736 for Random Forest; 0.971 and 0.559 for Gaussian Processes).

We also examined the performance of the Gaussian Processes model through cross-validation using bootstrap resampling (without replacement) in which 75% of the training data was used for model fitting and performance was evaluated on the remaining 25% OOB samples. Based on 1,000 bootstrap runs, the average error (for untransformed predictions of MWAT) was -0.0042 °C and the average absolute error was 0.588 °C – suggesting that the model fit is unbiased but somewhat imprecise. The average RMSE was 0.811, with empirical 95% confidence limits of 0.741 to 0.882 °C.

References

Butcher, J.B., D. Nover, T.E. Johnson, and C.M. Clark., (2015), Sensitivity of lake thermal and mixing dynamics to climate change, Climatic Change, doi:10.1007/s10584-015-1326-1.

Gorham, E., and F.M. Boyce, (1989), Influence of lake surface area and depth upon thermal stratification and the depth of the summer thermocline, J. Great Lakes Res., 15: 233–245.

Liaw, A. and M. Wiener, (2002), Classification and regression by randomForest, R News, 2: 18-22.

Neumann, M., S. Huang, D.E. Marthaler, and K. Kersting, (2015), pyGPS – A Python library for Gaussian process regression and classification, J. Mach. Learn. Res., 16: 2611-2616.

R Core Team, (2017), R: A language and environment for statistical computing. Vienna: R Foundation for Statistical Computing. https://www.R-project.org/.
