# Supplementary material for: Estimating future temperature maxima in lakes across the United States using a surrogate modeling approach
Source: PLoS One. 2017 Nov 9;12(11):e0183499. doi: 10.1371/journal.pone.0183499 (PMC5679518; doi:10.1371/journal.pone.0183499)
Supplement: S3 Fig — (DOCX) [file pone.0183499.s005.docx]

Supporting Information for

Estimates of Future Temperature Maxima in Lakes across the United States using a Surrogate Modeling Approach

Jonathan B. Butcher^1^, Tan Zi^2^, Michelle Schmidt^1^, Thomas E. Johnson^3^, Daniel M Nover^4^, and Christopher M. Clark^3^

^1^Tetra Tech, Inc., Research Triangle Park, NC; ^2^Tetra Tech, Inc., Fairfax, VA; ^3^ U.S. Environmental Protection Agency, Office of Research and Development, Washington, DC;
^4^ University of California – Merced, School of Engineering.

S3 Figure. Example Quasi-Random Sobol Sequence of 0-1 Triplets used to Create the LISSS Training Dataset
