# Supplementary material for: Estimating future temperature maxima in lakes across the United States using a surrogate modeling approach
Source: PLoS One. 2017 Nov 9;12(11):e0183499. doi: 10.1371/journal.pone.0183499 (PMC5679518; doi:10.1371/journal.pone.0183499)
Supplement: S1 Table — (DOCX) [file pone.0183499.s006.docx]

Supporting Information for

Estimates of Future Temperature Maxima in Lakes across the United States using a Surrogate Modeling Approach

Jonathan B. Butcher^1^, Tan Zi^2^, Michelle Schmidt^1^, Thomas E. Johnson^3^, Daniel M Nover^4^, and Christopher M. Clark^3^

^1^Tetra Tech, Inc., Research Triangle Park, NC; ^2^Tetra Tech, Inc., Fairfax, VA; ^3^ U.S. Environmental Protection Agency, Office of Research and Development, Washington, DC;
^4^ University of California – Merced, School of Engineering.

S1 Table. Identification of Meteorological Stations used in LISSS Training Dataset

| Station (COOP ID) | Name | Latitude | Longitude |
| --- | --- | --- | --- |
| FL088788 | TAMPA WSCMO AP, FL | 27.9614 | -82.5403 |
| NH275780 | NEW DURHAM 3 NNW, NH | 43.4833 | -71.1833 |
| MN218323 | TRACY, MN | 44.2394 | -95.6308 |
| MT244442 | ISMAY, MT | 46.4997 | -104.8 |
| AZ026323 | PAYSON, AZ | 34.2314 | -111.34 |
| OR352709 | EUGENE MAHLON SWEET FLD, OR | 44.1278 | -123.221 |
| CO058064 | SUGARLOAF RESERVOIR, CO | 39.2494 | -106.371 |
| TX412404 | DENTON, TX | 33.1989 | -97.105 |
| CA045114 | LOS ANGELES INTL AP, CA | 33.9381 | -118.389 |
