# Supplementary material for: Estimating future temperature maxima in lakes across the United States using a surrogate modeling approach
Source: PLoS One. 2017 Nov 9;12(11):e0183499. doi: 10.1371/journal.pone.0183499 (PMC5679518; doi:10.1371/journal.pone.0183499)
Supplement: S2 Table — (DOCX) [file pone.0183499.s007.docx]

Supporting Information for

Estimates of Future Temperature Maxima in Lakes across the United States using a Surrogate Modeling Approach

Jonathan B. Butcher^1^, Tan Zi^2^, Michelle Schmidt^1^, Thomas E. Johnson^3^, Daniel M Nover^4^, and Christopher M. Clark^3^

^1^Tetra Tech, Inc., Research Triangle Park, NC; ^2^Tetra Tech, Inc., Fairfax, VA; ^3^ U.S. Environmental Protection Agency, Office of Research and Development, Washington, DC;
^4^ University of California – Merced, School of Engineering.

S2 Table. GCMs and RCMs for NARCCAP Climate Scenarios

| Scenario | 0 | 1 | 2 | 3 | 4 | 5 | 6 |
| --- | --- | --- | --- | --- | --- | --- | --- |
| GCM | observed | CGCM3 | HadCM3 | GFDL | GFDL | CGCM3 | CCSM |
| RCM |  | CRCM | HRM3 | RCM3 | GFDL hi-res | RCM3 | WRFP |

GCMs

CGCM3: Third Generation Coupled Global Climate Model

http://www.ec.gc.ca/ccmac-cccma/default.asp?lang=En&n=4A642EDE-1

HADCM3: Hadley Centre Coupled Model, version 3

http://www-pcmdi.llnl.gov/ipcc/model_documentation/HadCM3.htm

GFDL: Geophysical Fluid Dynamics Laboratory GCM

http://www-pcmdi.llnl.gov/ipcc/model_documentation/GFDL-cm2.htm

CCSM: Community Climate System Model

http://www-pcmdi.llnl.gov/ipcc/model_documentation/CCSM3.htm

RCMs

CRCM: Canadian Regional Climate Model

http://www.ec.gc.ca/ccmac-cccma/default.asp?lang=En&n=4A642EDE-1

RCM3: Regional Climate Model, version 3

http://users.ictp.it/~pubregcm/RegCM3/

HRM3: Hadley Region Model 3

http://precis.metoffice.com/

WRFG: Weather Research and Forecasting Model

http://www.wrf-model.org/index.php

GFDL hi-res: Geophysical Fluid Dynamics Laboratory 50-km global atmospheric timeslice

http://www-pcmdi.llnl.gov/ipcc/model_documentation/GFDL-cm2.htm
