# Supplementary material for: Estimating future temperature maxima in lakes across the United States using a surrogate modeling approach
Source: PLoS One. 2017 Nov 9;12(11):e0183499. doi: 10.1371/journal.pone.0183499 (PMC5679518; doi:10.1371/journal.pone.0183499)
Supplement: S3 Table — (DOCX) [file pone.0183499.s008.docx]

Supporting Information for

Estimates of Future Temperature Maxima in Lakes across the United States using a Surrogate Modeling Approach

Jonathan B. Butcher^1^, Tan Zi^2^, Michelle Schmidt^1^, Thomas E. Johnson^3^, Daniel M Nover^4^, and Christopher M. Clark^3^

^1^Tetra Tech, Inc., Research Triangle Park, NC; ^2^Tetra Tech, Inc., Fairfax, VA; ^3^ U.S. Environmental Protection Agency, Office of Research and Development, Washington, DC;
^4^ University of California – Merced, School of Engineering.

S3 Table. Correlation Coefficients for the Simulations of MWAT above the Thermocline in *Butcher et al.* [2015]

|  | MWAT | July/Aug Atemp | Jan Atemp | Depth (m) | Area (m^2^) | Extinction (m^-1^) | PET |
| --- | --- | --- | --- | --- | --- | --- | --- |
| MWAT | 1.00 |  |  |  |  |  |  |
| July/Aug Atemp | 0.83 | 1.00 |  |  |  |  |  |
| Jan Atemp | 0.55 | 0.45 | 1.00 |  |  |  |  |
| depth(m) | -0.22 | < 0.01 | < 0.01 | 1.00 |  |  |  |
| SA (m^2^) | -0.23 | < 0.01 | < 0.01 | < 0.01 | 1.00 |  |  |
| Extinction (m^-1^) | -0.16 | < 0.01 | < 0.01 | < 0.01 | < 0.01 | 1.00 |  |
| PET | 0.64 | 0.86 | 0.54 | < 0.01 | < 0.01 | < 0.01 | 1.00 |

Reference

Butcher, J.B., D. Nover, T.E. Johnson, and C.M. Clark., (2015), Sensitivity of lake thermal and mixing dynamics to climate change, Climatic Change, doi:10.1007/s10584-015-1326-1.
