# Supplementary material for: Estimating future temperature maxima in lakes across the United States using a surrogate modeling approach
Source: PLoS One. 2017 Nov 9;12(11):e0183499. doi: 10.1371/journal.pone.0183499 (PMC5679518; doi:10.1371/journal.pone.0183499)
Supplement: S4 Table — (DOCX) [file pone.0183499.s009.docx]

Supporting Information for

Estimates of Future Temperature Maxima in Lakes across the United States using a Surrogate Modeling Approach

Jonathan B. Butcher^1^, Tan Zi^2^, Michelle Schmidt^1^, Thomas E. Johnson^3^, Daniel M Nover^4^, and Christopher M. Clark^3^

^1^Tetra Tech, Inc., Research Triangle Park, NC; ^2^Tetra Tech, Inc., Fairfax, VA; ^3^ U.S. Environmental Protection Agency, Office of Research and Development, Washington, DC;
^4^ University of California – Merced, School of Engineering.

S4 Table. AIC Obtained from Optimizing Seven Covariance Kernel Functions to Training Data

| Covariance Function | AIC |
| --- | --- |
| 1. Squared exponential kernel | 233 |
| 1. Linear kernel | 4,218 |
| 1. Polynomial kernel | 745 |
| 1. Rational quadratic kernel | 127 |
| 1. Squared exponential + rational quadratic kernel | 99 |
| 1. Matern kernel | 1,768 |
| 1. Squared exponential kernel with unit magnitude | 239 |
