# Supplementary material for: Estimating future temperature maxima in lakes across the United States using a surrogate modeling approach
Source: PLoS One. 2017 Nov 9;12(11):e0183499. doi: 10.1371/journal.pone.0183499 (PMC5679518; doi:10.1371/journal.pone.0183499)
Supplement: S5 Table — (DOCX) [file pone.0183499.s010.docx]

Supporting Information for

Estimates of Future Temperature Maxima in Lakes across the United States using a Surrogate Modeling Approach

Jonathan B. Butcher^1^, Tan Zi^2^, Michelle Schmidt^1^, Thomas E. Johnson^3^, Daniel M Nover^4^, and Christopher M. Clark^3^

^1^Tetra Tech, Inc., Research Triangle Park, NC; ^2^Tetra Tech, Inc., Fairfax, VA; ^3^ U.S. Environmental Protection Agency, Office of Research and Development, Washington, DC;
^4^ University of California – Merced, School of Engineering.

S5 Table. Optimized Posterior Hyper-parameters for Selected Covariance Model (Sum of rational quadratic kernel and squared exponential kernel)

| Parameter | Rational Quadratic Component | Squared Exponential Component |
| --- | --- | --- |
| Standard deviation of noise fluctuation (*σ*) | 1.676 | 2.731 |
| Characteristic Length (*l*) | 0.0844 | 0.268 |
| Shape Parameter (*α)* | -2.059 | NA |
| Final negative log marginal likelihood | 44.255 | |
